# Art an 3 as a candidate recombinant antigen for Artemisia pollen immunotherapy: comparative efficacy and mechanistic insights

**DOI:** 10.3389/fmed.2026.1864362

**Published:** 2026-07-22

**Authors:** Jiacheng Shen, Tao Wang, Bin Zhou, Bowen Tian, Binhao Liu, Jiachen Gui, Yue Zhang, Wenzhi Hu, Yuting Lai, Honghao Zhang, Qiang Li

**Affiliations:** 1 The Air Force Clinical College, Anhui Medical University, Hefei, Anhui, China; 2 Department of Dermatology, West China Hospital, Sichuan University, Chengdu, Sichuan, China; 3 Department of Dermatology, Mianyang 404 Hospital, Mianyang, China; 4 Department of Dermatology, The Fourth Affiliated Hospital of Soochow University, Suzhou Dushu Lake Hospital, Suzhou, Jiangsu, China; 5 Graduate School, Air Force Medical University, Xi’an, China; 6 Department of Dermatology, 960th Hospital, PLA, Jinan, Shandong, China; 7 Hospital of Unit 93926, PLA, Hotan, Xinjiang, China; 8 Department of Dermatology, Air Force Medical Center, PLA, Beijing, China; 9 Department of Burn and Plastic Surgery, Air Force Medical Center, PLA, Beijing, China; 10 Graduate School, Hebei North University, Zhangjiakou, Hebei, China

**Keywords:** airway inflammation, allergen-specific immunotherapy, allergy, *Artemisia annua*, immune tolerance induction, recombinant protein

## Abstract

**Background:**

Artemisia pollen is a major cause of seasonal respiratory allergy, yet allergen-specific immunotherapy (AIT) still relies mainly on crude extracts. Art an 3 is a major *Artemisia annua* pollen allergen, but its therapeutic and immunological features relative to extracts remain unclear.

**Objective:**

To compare recombinant Art an 3 (rArt an 3)–based versus extract-based subcutaneous immunotherapy (SCIT) in an *A. annua* pollen–induced murine asthma model.

**Methods:**

Female BALB/c mice were sensitized and intranasally challenged with *A. annua* pollen extract, then treated with Extracts-SCIT, rArt an 3-SCIT, or PBS. Airway hyperresponsiveness (AHR), lung histopathology, bronchoalveolar lavage fluid (BALF) cells, splenic cytokines, serum immunoglobulins, and splenic CD4^+^ T-cell subsets were evaluated.

**Results:**

Both SCIT regimens reduced peribronchial inflammation, goblet-cell mucus, and methacholine-induced AHR versus positive controls; histologic improvement tended to be greater with Extracts-SCIT, whereas AHR improvement was comparable. Extracts-SCIT more clearly decreased BALF eosinophils and IL-5/IL-17A. rArt an 3-SCIT preferentially increased IL-10 and IFN-*γ* and enhanced splenic Treg/Th1 responses. Pollen-specific sIgE decreased most with Extracts-SCIT, while rArt an 3-SCIT showed a modest sIgE reduction but a more pronounced increase in pollen-specific sIgG1.

**Conclusion:**

rArt an 3-SCIT showed overall protection comparable to Extracts-SCIT, with a regulatory/Th1-associated profile characterized by increased Treg/Th1 responses and pollen-specific sIgG1, supporting rArt an 3 as a molecularly defined AIT candidate for Artemisia pollen allergy.

## Introduction

IgE-mediated allergic diseases have become a major global public health burden (1). For respiratory allergies in particular, it is estimated that approximately 300 million people worldwide suffer from allergic asthma and 500 million from allergic rhinitis (2). Allergen-specific immunotherapy (AIT) is currently the only disease-modifying treatment capable of altering the natural course of allergic disease (3–5). Conventional AIT vaccines used in the clinic are mostly based on crude natural allergen extracts, whose complex composition, batch-to-batch variability and limited standardization require stringent quality control and risk management during use (6–10). Immunotherapy with recombinant allergens can induce allergen-specific IgG “blocking antibodies” that compete with IgE for allergen-binding epitopes, thereby inhibiting effector-cell activation and alleviating allergic inflammation (11). Previous studies have further indicated that highly purified, standardized recombinant allergens reduce interference from non-specific impurities, allow more accurate dose definition and provide a more controllable system for mechanistic investigations (12–14). In many regions worldwide, pollen from Artemisia species is among the most common causes of seasonal respiratory allergy, including allergic rhinitis and asthma (15). A survey of 13 cities in northern China by Zhang et al. showed that Artemisia pollen is the predominant airborne allergenic pollen in autumn, accounting for up to 72.31% of annual airborne pollen counts in some north-western areas (16). In Europe, Artemisia pollen is likewise a major seasonal aeroallergen, with sensitization rates generally around 10–14% (17) and reaching up to 44% in highly exposed regions (18).

Art an 3 is one of the major pollen allergens identified in *Artemisia annua*, classified as a non-specific lipid transfer protein (nsLTP). According to the WHO/IUIS allergen database, recombinant Art an 3 (rArt an 3) is specifically recognized by serum IgE from a subset of pollen-allergic patients (*n* = 28, 14 positive). Wang et al. further demonstrated that rArt an 3 is recognized by patients’ IgE and can compete with crude Artemisia extract for IgE binding, indicating clear IgE-binding reactivity and supporting its potential as a target for immunological intervention (19). However, the mechanisms by which Art an 3 contributes to immune tolerance remain poorly characterized, and head-to-head comparisons with conventional extracts-based AIT are still lacking.

In the present study, we generated a recombinant Art an 3 protein (rArt an 3) and evaluated its therapeutic potential in a murine model of *A. annua* pollen-induced asthma using subcutaneous immunotherapy (SCIT). By directly comparing rArt an 3-SCIT with Extracts-SCIT, we sought to delineate the ability of rArt an 3 to induce immune tolerance, reshape humoral immune responses and ameliorate airway inflammation, thereby providing preclinical evidence supporting rArt an 3 AIT for Artemisia-induced allergy.

## Materials and methods

### Preparation of *Artemisia annua* pollen extract

Preparation of *A. annua* pollen extract was performed following a previously described protocol (20). Briefly, pollen was ground in liquid nitrogen, defatted with acetone, extracted with 0.125 M ammonium bicarbonate, dialyzed and lyophilized. The protein concentration of the resulting crude extract was determined using a bicinchoninic acid (BCA) assay.

### Expression, purification, and IgE immunoreactivity of recombinant Art an 3

The Art an 3 gene (GenBank: KR996740) was codon-optimized and cloned into the pSmart-I expression vector to generate N-terminal SUMO fusion protein containing a polyhistidine purification motif. The construct was expressed in *E. coli* BL21 (DE3). When cultures reached an OD600 of 0.6–0.8, protein expression was induced with IPTG. Bacterial pellets were collected and lysed by sonication in buffer containing 20 mM phosphate buffer (PB) and 150 mM NaCl (pH 8.0). After clarification through a 0.22-μm filter, the lysate was loaded onto a Ni-NTA affinity column, and the eluted protein was dialyzed into storage buffer. Protein expression and purity were assessed by SDS-PAGE followed by Coomassie Brilliant Blue staining. Endotoxin contamination in the purified recombinant protein preparation was assessed using a gel-clot Limulus amebocyte lysate assay with a sensitivity of 0.25 EU/mL.

To evaluate IgE-binding activity, sera from five Artemisia-allergic individuals and five healthy controls were obtained from archived hospital serum samples and pooled to generate IgE-positive and IgE-negative serum pools, respectively. All human serum samples were de-identified prior to use. Purified rArt an 3 was transferred to PVDF membranes after SDS-PAGE and probed with either the allergic or healthy serum pool, followed by HRP-conjugated anti-human IgE for detection.

### Animals

Female BALB/c mice (6–8 weeks old, 16–20 g) were purchased from Beijing SPF Biotechnology Co., Ltd. (Beijing, China) and housed under specific pathogen-free (SPF) conditions. This strain and sex were selected to maintain consistency with commonly used Th2-prone murine allergic asthma models and to reduce sex-related biological variability during comparison of the two SCIT regimens (21, 22). Mice were maintained at 23–25 °C with 50–60% relative humidity under a 12-h light/dark cycle, with food and water available ad libitum. All *in vivo* animal experiments were performed in an accredited SPF animal facility. This study was conducted and reported in accordance with the ARRIVE guidelines for reporting animal research (23). At the experimental endpoint, mice were euthanized by isoflurane inhalation overdose (5%), followed by cervical dislocation to ensure death. All procedures were performed in accordance with the American Veterinary Medical Association (AVMA) Guidelines for the Euthanasia of Animals.

### Sensitization, challenge and specific immunotherapy

A total of 40 female BALB/c mice (6–8 weeks) were randomly assigned to four groups (*n* = 10 per group) before sensitization: negative control (NC), positive control (PC), crude extract SCIT (Extracts-SCIT) and recombinant protein SCIT (rArt an 3-SCIT). The group size was determined with reference to previous murine allergic asthma and SCIT studies, while also considering ethical principles for animal use and experimental feasibility (24, 25). Except for the NC group, all mice were sensitized subcutaneously on days 1, 8, 15 and 22 with 50 μg *A. annua* pollen extract adsorbed to 2 mg aluminum hydroxide in 200 μL PBS. On days 29–31, mice were challenged intranasally with 200 μg *A. annua* pollen extract in 20 μL PBS once daily for three consecutive days. NC mice received PBS alone at the same time points.

Beginning on day 34, mice underwent four weekly SCIT injections (days 34, 41, 48 and 55). The Extracts-SCIT group received 50 μg crude pollen extract in 200 μL PBS, whereas the rArt an 3-SCIT group received 50 μg rArt an 3 in 200 μL PBS. NC and PC mice were injected with equal volumes of PBS to control for handling and injection effects.

**Figure 1:**
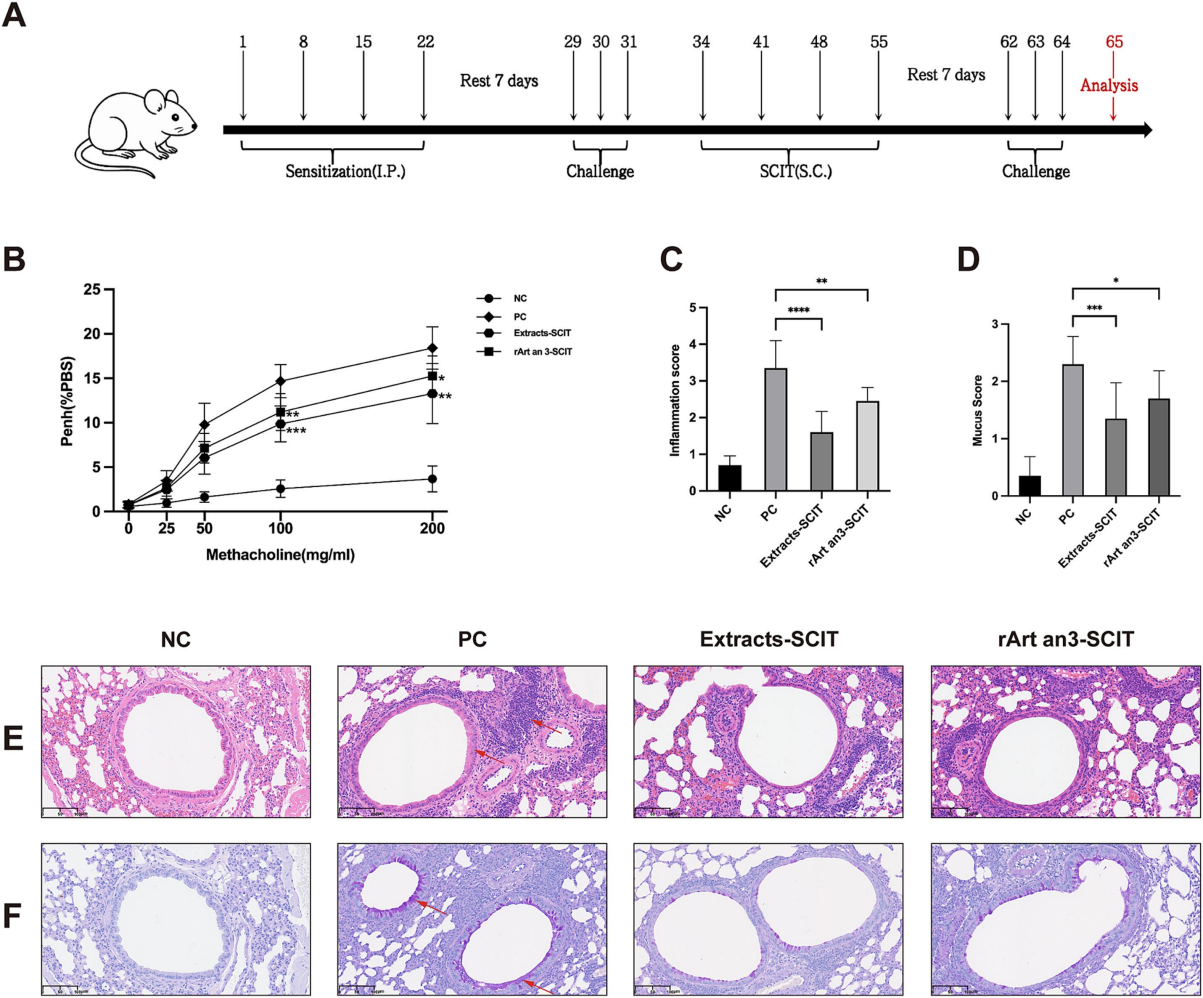
Effects of Extracts-SCIT and rArt an 3-SCIT on airway pathology and airway hyperresponsiveness in *Artemisia annua* pollen-induced asthma. **(A)** Schematic of the sensitization, challenge and subcutaneous immunotherapy (SCIT) protocol. Female BALB/c mice were sensitized with *A. annua* pollen extract, intranasally challenged, and treated with four weekly SCIT injections of crude extract (Extracts-SCIT) or recombinant Art an 3 (rArt an 3-SCIT), as indicated. **(B)** Airway hyperresponsiveness assessed by Penh in response to aerosolized methacholine (0–200 mg/mL). **(C)** Semi-quantitative lung inflammation scores based on peribronchial and perivascular inflammatory infiltrates in H&E-stained sections. **(D)** Semi-quantitative mucus scores based on goblet-cell hyperplasia and epithelial mucus in PAS-stained sections. **(E)** H&E-stained lung sections from NC, PC, Extracts-SCIT and rArt an 3-SCIT groups. Scale bar = 100 μm. **(F)** PAS-stained lung sections from the same groups showing airway mucus production. Scale bar = 100 μm. Data in **(B–D)** are presented as mean ± SD (n = 10 per group). Statistical significance in **(B)** was determined by two-way ANOVA followed by Tukey’s multiple comparisons test, whereas statistical significance in **(C,D)** was determined by one-way ANOVA followed by Tukey’s multiple comparisons test. * *p* < 0.05, ** *p* < 0.01, *** *p* < 0.001 vs. PC group.

On days 62–64, all groups except NC were re-challenged intranasally with 200 μg *A. annua* pollen extract (NC: PBS). Mice were sacrificed on day 65 and bronchoalveolar lavage fluid (BALF), lung tissues and spleens were collected for downstream analyses (Figure 1A; Table 1).

**Table 1:** Experimental design of sensitization, challenge, and subcutaneous immunotherapy (SCIT) in the *Artemisia annua*–induced asthma model.

Group	Sensitization (Days 1–22)	Challenge (Days 29–31)	SCIT (Days 34–55)	Re-challenge (Days 62–64)
NC	200 μL, PBS	20 μL, PBS	200 μL, PBS	20 μL, PBS
PC	200 μL, 50 μg Extracts + 2 mg Alum	20 μL, 200 μg Extracts	200 μL, PBS	20 μL, 200 μg Extracts
Extracts-SCIT	200 μL, 50 μg Extracts + 2 mg Alum	20 μL, 200 μg Extracts	200 μL, 50 μg Extracts	20 μL, 200 μg Extracts
rArt an 3-SCIT	200 μL, 50 μg Extracts + 2 mg Alum	20 μL, 200 μg Extracts	200 μL, 50 μg rArt an 3	20 μL, 200 μg Extracts

NC, negative control; PC, positive control; Extracts, *Artemisia annua* pollen extract; SCIT, subcutaneous immunotherapy; rArt an 3, recombinant *Artemisia annua* allergen Art an 3; PBS, phosphate-buffered saline; Alum, aluminum.

### Assessment of airway hyperresponsiveness

Airway hyperresponsiveness (AHR) was assessed on day 65 using whole-body plethysmography (WBP) in conscious, unrestrained mice. Following established protocols (26), airway obstruction was quantified by the enhanced pause (Penh). Mice were placed in a sealed chamber and first exposed to aerosolized PBS for 5 min to obtain baseline values, followed by nebulized methacholine at 25, 50, 100 and 200 mg/mL. Penh values were recorded at each concentration.

### Collection of BALF and inflammatory cell counts

Following euthanasia, mice were placed in a supine position and the trachea was exposed by gentle dissection. A sterile cannula was inserted into the trachea, and the lungs were lavaged five times with sterile PBS. The recovered lavage fluid was centrifuged at 1400 rpm for 5 min, the supernatant was removed, and the cell pellet was resuspended in PBS. Total leukocytes and selected leukocyte populations in BALF, including eosinophils, neutrophils, and mononuclear cells, were quantified using an automated hematology analyzer (Sysmex XN-1000, Kobe, Japan).

### Preparation of spleen homogenates and cytokine measurement

Spleens were aseptically excised, cleared of adherent connective tissue and thoroughly rinsed in ice-cold PBS to remove residual blood. Each spleen was homogenized in PBS at a 1:9 (w/v) tissue-to-buffer ratio using a mechanical homogenizer. The homogenates were centrifuged at 12,000 rpm for 10 min at 4 °C, and the resulting supernatants were collected for cytokine analysis. Levels of IL-4, IL-5, IL-10, IL-17A, IFN-*γ* and TGF-β1 were measured using commercial enzyme-linked immunosorbent assay (ELISA) kits (Thermo Fisher Scientific) according to the manufacturer’s instructions. Absorbance was recorded at 450 nm, and cytokine concentrations were calculated from standard curves.

### Flow cytometric analysis of T-cell subsets

Single-cell suspensions were generated from spleens by mechanical dissociation followed by red blood cell lysis. Cells were stained with fluorochrome-conjugated antibodies against CD3, CD4, CD25 and CXCR5 (CD185), then fixed and permeabilized for intracellular staining of Foxp3, IL-4 and IFN-*γ*. Data were acquired on a Beckman CytoFLEX S flow cytometer and analyzed using FlowJo v10. Compensation was performed using single-stained controls. Fluorescence gates were set with reference to unstained controls and corresponding single-stained controls.

For gating, lymphocytes were first identified based on forward scatter and side scatter characteristics, followed by exclusion of cell aggregates using FSC-A/FSC-H gating. CD4^+^ T cells were then selected as the parent population for Treg and Tfh-like cell analysis. Treg cells were defined as CD25^+^Foxp3^+^ cells within the CD4^+^ T-cell population, and Tfh-like cells were defined as CXCR5^+^ cells within the CD4^+^ T-cell population. For intracellular cytokine analysis, CD3^+^CD4^+^ T cells were gated prior to assessment of IFN-*γ* and IL-4 expression. Th1 and Th2 cells were defined as IFN-γ^+^ and IL-4^+^ cells within the CD3^+^CD4^+^ T-cell population, respectively. Representative gating plots are provided in Supplementary Figure S2.

### Measurement of serum antibodies

Whole blood was collected at sacrifice and centrifuged at 4 °C, 1000 rpm for 20 min to obtain serum, which was stored at −80 °C until analysis. Total IgE and total IgG levels were quantified using commercial ELISA kits (Thermo Fisher Scientific) following the manufacturer’s instructions. *A. annua* pollen–specific sIgE, sIgG1 and sIgG2a were measured using in-house ELISAs. *A. annua* pollen extract was coated onto 96-well plates as the solid-phase antigen, followed by blocking and incubation with diluted serum samples at 37 °C for 2 h. After washing, biotinylated secondary antibodies and HRP-conjugated streptavidin were added sequentially for color development. Absorbance at 450 nm was recorded. Specific antibody responses were expressed as relative OD450 values, whereas total antibody concentrations were calculated from standard curves.

### Histopathology

Left lungs were excised immediately after sacrifice and fixed in 4% paraformaldehyde (pH 7.4) for 24 h, followed by routine dehydration, clearing and paraffin embedding. Serial 4-μm sections were prepared. Hematoxylin–eosin (HE) staining was performed to evaluate inflammatory cell infiltration around bronchi and blood vessels, and periodic acid–Schiff (PAS) staining was used to assess goblet-cell hyperplasia and airway epithelial mucus production. Lung inflammation and mucus secretion were semi-quantitatively scored according to published methods (27–29). Inflammatory cell infiltration and PAS-positive mucus were each graded from 0 to 4 based on the extent and intensity of lesions. Histological scoring was performed by investigators blinded to group allocation. For each mouse, individual scores were summarized and expressed as mean ± SD.

### Statistical analysis

Statistical analyses were performed using GraphPad Prism 10.0.2 (GraphPad Software, San Diego, CA, United States). Data are presented as mean ± standard deviation (SD). Normality was assessed using the Shapiro–Wilk test, and homogeneity of variance was evaluated using Levene’s test. For normally distributed data with equal variances, one-way ANOVA followed by Tukey’s *post hoc* test was used for multiple-group comparisons; otherwise, the Kruskal–Wallis test with Dunn’s multiple comparisons was applied. Penh–methacholine dose–response curves were analyzed using two-way ANOVA with Tukey’s post hoc test. Outcome measurements were analyzed using coded sample identifiers, with group allocation concealed during data analysis. A *p* value < 0.05 was considered statistically significant.

## Results

### Expression, purity, and IgE reactivity of recombinant Art an 3

**Figure 2:**
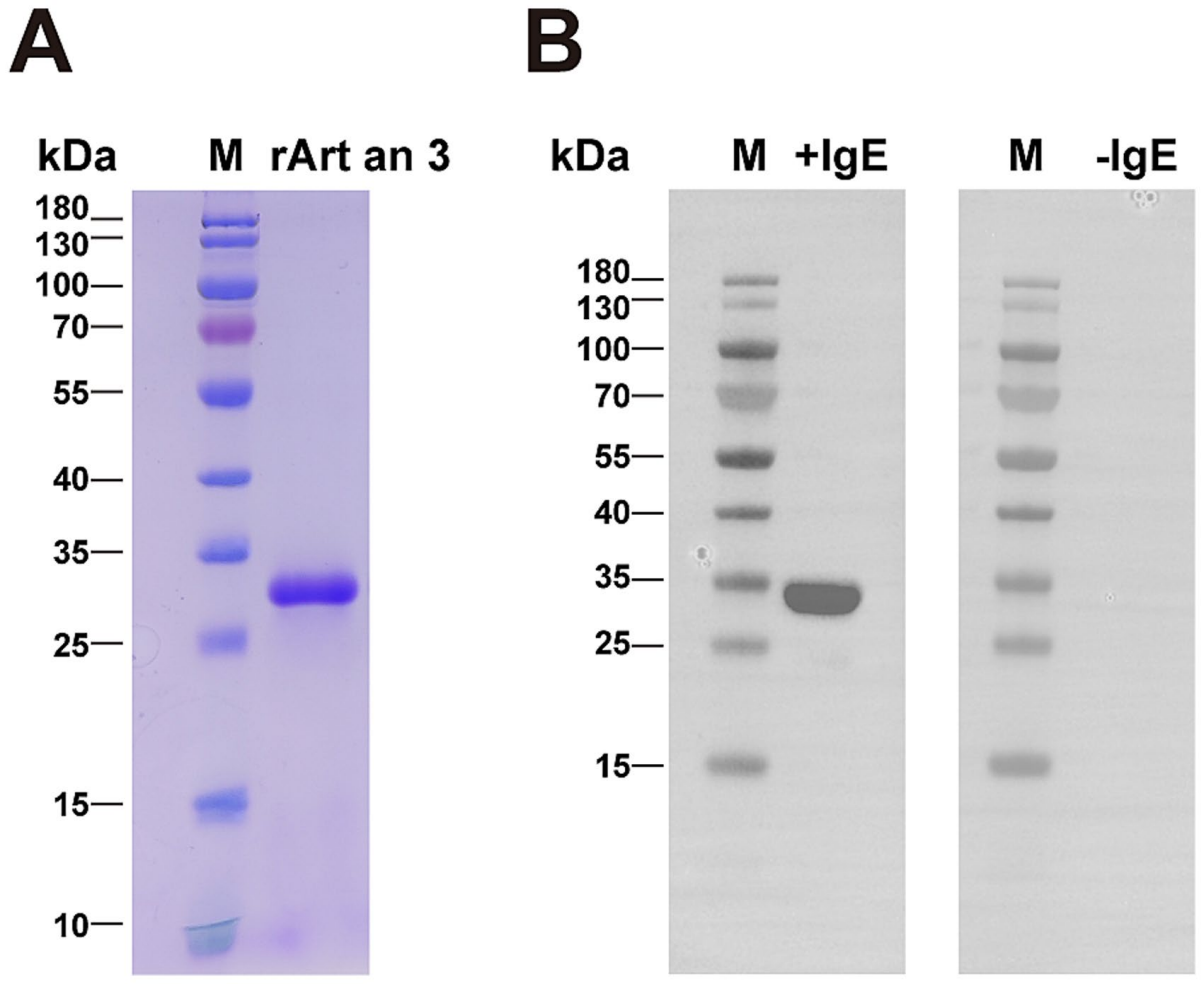
Expression, purification, and IgE immunoreactivity of recombinant Art an 3. **(A)** Coomassie Brilliant Blue–stained SDS-PAGE analysis of purified rArt an 3. lane M, molecular weight marker; lane rArt an 3, purified recombinant Art an 3 fusion protein (~25–30 kDa). **(B)** IgE immunoblotting of purified rArt an 3. lane +IgE: rArt an 3 probed with IgE-positive serum pool from Artemisia pollen–allergic individuals; lane –IgE: rArt an 3 probed with IgE-negative serum from healthy controls; lane M: molecular weight marker.

Recombinant Art an 3 was expressed in *E. coli* as an N-terminal SUMO fusion protein containing a polyhistidine purification motif and purified by Ni-NTA affinity chromatography. SDS-PAGE analysis revealed a single, well-defined band at ~30 kDa (Figure 2A), consistent with the predicted molecular weight of the SUMO–Art an 3 fusion protein containing the polyhistidine purification motif. Because native Art an 3 is ~10 kDa (30), the higher apparent molecular mass reflects the additional SUMO fusion partner (31). The purified protein showed >90% purity. Endotoxin testing showed no gel clot formation in the recombinant protein sample at 1 mg/mL, indicating that the endotoxin level was below the assay sensitivity (<0.25 EU/mL), corresponding to <0.25 EU/mg protein (Supplementary Figure S1).

IgE immunoblotting demonstrated a clear positive band at ~30 kDa when probed with the IgE-positive serum pool, whereas no band was detected using IgE-negative serum from healthy donors (Figure 2B). These findings confirm that the recombinant fusion protein retained IgE-binding reactivity and was recognized by IgE from Artemisia-allergic individuals, indicating preservation of IgE-recognized antigenic determinants.

### Establishment of the *Artemisia annua* pollen–induced asthma model

**Figure 3:**
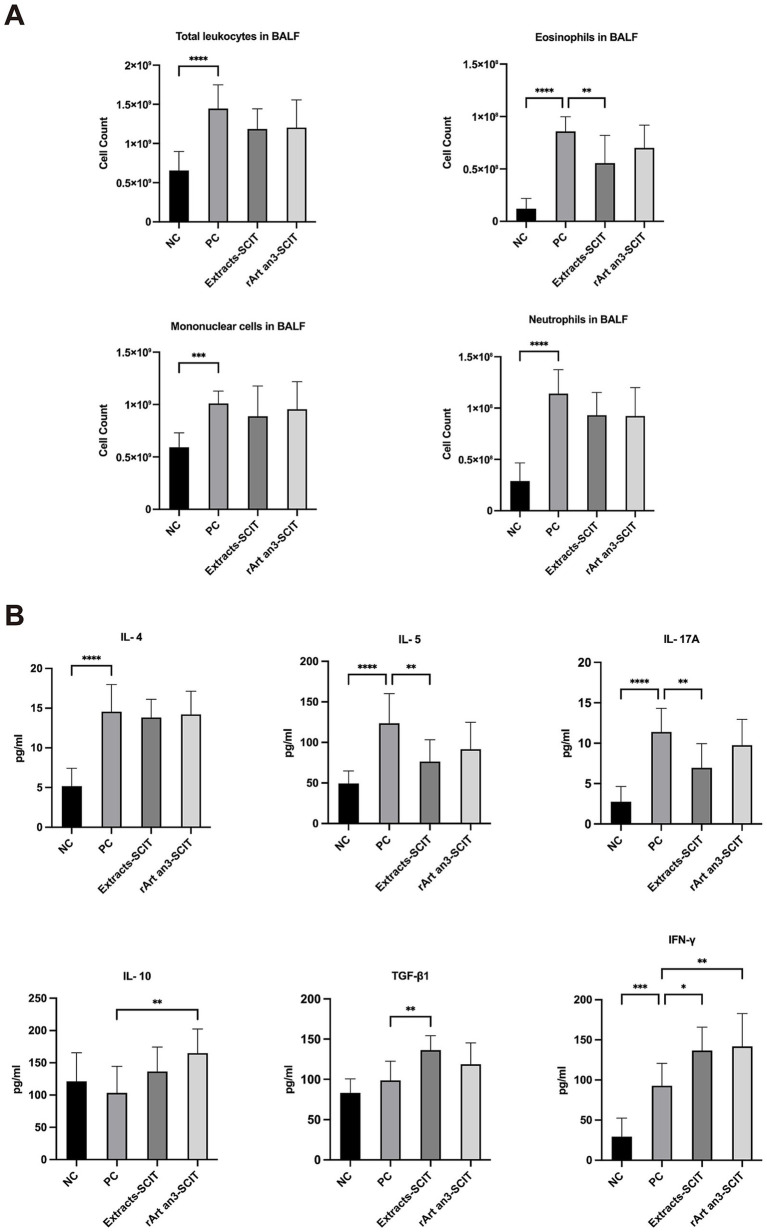
Differential effects of Extracts-SCIT and rArt an 3-SCIT on spleen cytokines and BALF inflammatory cell profiles. **(A)** Bronchoalveolar lavage fluid (BALF) total leukocyte, eosinophil, mononuclear cell, and neutrophil counts. **(B)** Concentrations of IL-4, IL-5, IL-17A, IL-10, TGF-β1, and IFN-*γ* in spleen homogenates from the NC, PC, Extracts-SCIT, and rArt an 3-SCIT groups. Cytokines were measured by ELISA and are expressed as pg./mL. Data are presented as mean ± SD (*n* = 10 per group). Statistical significance was determined by one-way ANOVA followed by Tukey’s multiple comparisons test. * *p* < 0.05, ** *p* < 0.01, *** *p* < 0.001, **** *p* < 0.0001 vs. PC group.

**Figure 4:**
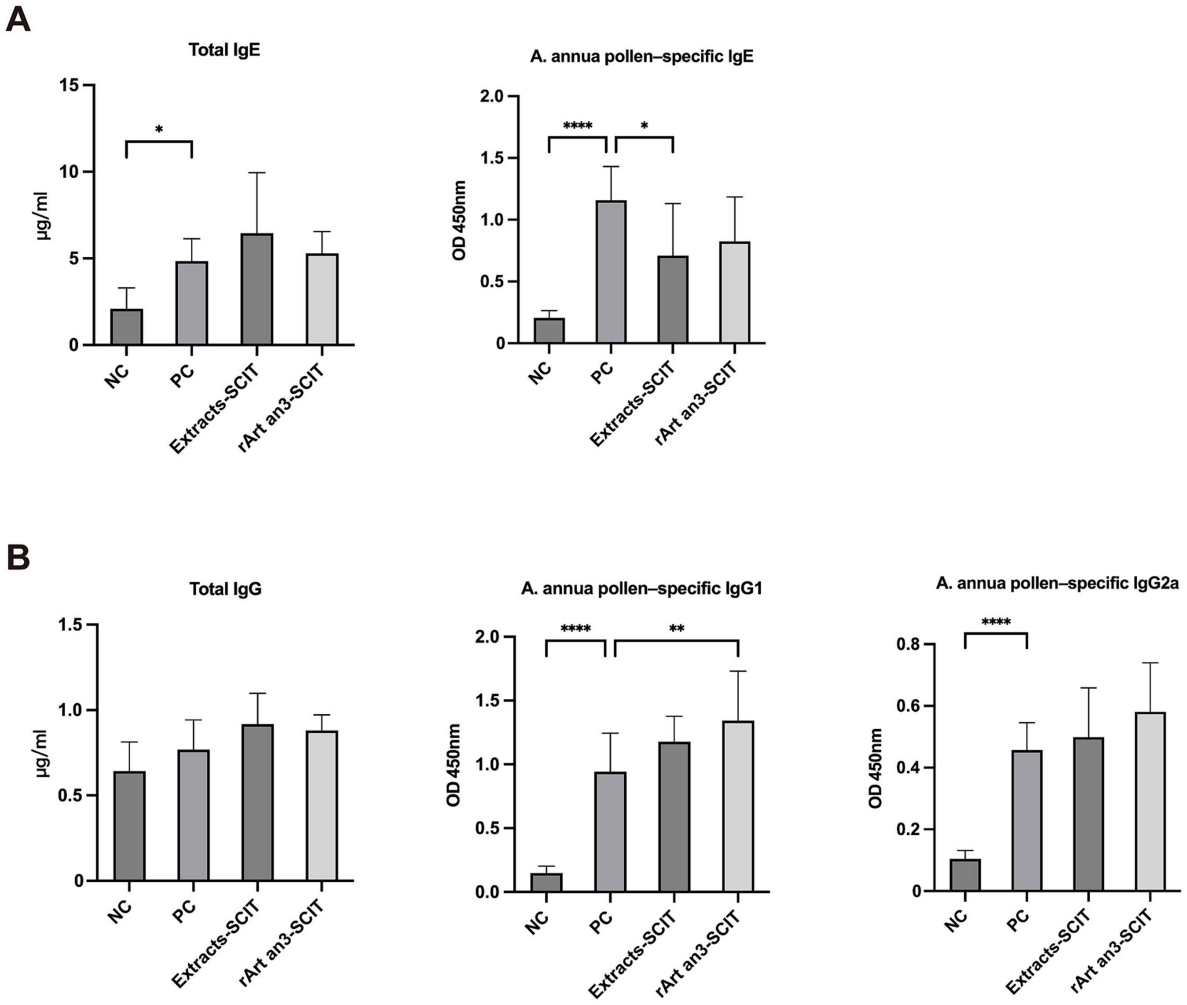
Effects of Extracts-SCIT and rArt an 3-SCIT on total and *Artemisia annua* pollen-specific immunoglobulins. **(A)** Total serum IgE and *A. annua* pollen-specific IgE (sIgE) levels in the NC, PC, Extracts-SCIT, and rArt an 3-SCIT groups. **(B)** Total serum IgG and *A. annua* pollen-specific IgG1 and IgG2a levels in the NC, PC, Extracts-SCIT, and rArt an 3-SCIT groups. Data are presented as mean ± SD (*n* = 10 per group). * *p* < 0.05, ** *p* < 0.01, *** *p* < 0.001, **** *p* < 0.0001 vs. PC group.

NC mice exhibited normal lung architecture, with minimal peribronchial or perivascular inflammatory infiltration and scant goblet-cell mucus production. BALF total leukocyte counts and serum IgE levels remained low. In contrast, PC mice developed a typical allergic asthma phenotype, characterized by significantly higher lung inflammation scores and markedly increased goblet-cell mucus secretion (Figures 1C,D). Moreover, BALF total leukocyte, eosinophil, neutrophil, and mononuclear cell counts were all significantly increased in the PC group compared with the NC group (*p* < 0.001; Figure 3A). Serum *A. annua* pollen-specific IgE levels were also significantly elevated relative to those in NC mice (*p* < 0.05; Figure 4A). These findings confirm the successful establishment of the *A. annua* pollen-induced asthma model.

### rArt an 3-SCIT alleviates airway inflammation and mucus hypersecretion

Compared with PC mice, both SCIT regimens attenuated lung inflammation and mucus production. HE staining revealed dense peribronchial and perivascular inflammatory infiltrates in PC mice, whereas inflammatory cell accumulation was reduced in both Extracts-SCIT and rArt an 3-SCIT groups (Figure 1E). PAS staining further showed pronounced goblet-cell hyperplasia and mucus hypersecretion in the PC group, while SCIT treatment led to decreased PAS-positive staining (Figure 1F). Consistently, semi-quantitative inflammation and mucus scores were significantly lower in both SCIT groups than in PC mice (*p* < 0.05; Figures 1C,D). Although Extracts-SCIT showed numerically greater reductions than rArt an 3-SCIT, the difference between the two regimens was not statistically significant.

### rArt an 3-SCIT improves airway hyperresponsiveness

Airway hyperresponsiveness was evaluated by Penh responses to escalating doses of methacholine (25, 50, 100, and 200 mg/mL). As shown in Figure 1B, PC mice displayed a dose-dependent rise in Penh relative to NC mice, indicating airway hyperresponsiveness. Both SCIT regimens significantly lowered Penh values at multiple methacholine concentrations (*p* < 0.05, *p* < 0.01 and *p* < 0.001, respectively). Although the Extracts-SCIT group showed numerically lower Penh values than the rArt an 3-SCIT group across doses, the difference between the two treatments did not reach statistical significance. Data were analyzed using two-way ANOVA with Tukey’s *post hoc* test and are presented as mean ± SD.

### rArt an 3-SCIT reshapes BALF inflammatory cell profiles and airway cytokine patterns

Regarding BALF cellular composition, eosinophil counts were significantly decreased in the Extracts-SCIT group compared with PC mice (*p* < 0.01), whereas the rArt an 3-SCIT group showed a non-significant decrease (Figure 3A). Total leukocyte, neutrophil, and mononuclear cell counts showed non-significant decreases in both treatment groups compared with PC mice.

At the cytokine level, IL-5 and IL-17A concentrations in PC mice were significantly elevated compared with NC mice (*p* < 0.01). IL-10 did not differ significantly between PC and NC mice, whereas TGF-β1 and IFN-*γ* showed non-significant increases in PC mice (Figure 3B). Following SCIT, IL-5 and IL-17A were significantly reduced in the Extracts-SCIT group compared with PC mice (*p* < 0.01), whereas the rArt an 3-SCIT group showed non-significant decreases in these cytokines. IL-10 was significantly elevated only in the rArt an 3-SCIT group compared with PC mice (*p* < 0.01). TGF-β1 increased significantly in the Extracts-SCIT group compared with PC mice (*p* < 0.01), while the change in the rArt an 3-SCIT group did not reach statistical significance. Both SCIT regimens significantly enhanced IFN-γ levels (*p* < 0.05), with no significant difference between the two treatment groups. IL-4 levels did not differ significantly among groups.

### rArt an 3-SCIT adjusts serum immunoglobulin responses

Total IgE, total IgG and *A. annua* pollen–specific sIgE, sIgG1 and sIgG2a were quantified (Figure 4). Compared with NC mice, the PC group showed a marked increase in sIgE (*p* < 0.05). Following SCIT, sIgE levels were significantly reduced in the Extracts-SCIT group relative to PC (*p* < 0.05), whereas the rArt an 3-SCIT group showed a non-significant decrease. Total IgE was higher in PC than in NC mice, and neither SCIT regimen induced a significant decrease compared with PC.

For IgG responses, pollen-specific sIgG1 was elevated in PC mice relative to NC. SCIT further enhanced sIgG1 production, with a significant increase observed in the rArt an 3-SCIT group compared with PC mice (*p* < 0.01), while the Extracts-SCIT group showed a non-significant elevation. Pollen-specific sIgG2a levels were numerically higher in both SCIT groups than in NC mice; however, neither treatment differed significantly from PC mice. Total IgG levels remained comparable across all groups.

### rArt an 3-SCIT modulates splenic CD4^+^ T-cell subsets

**Figure 5:**
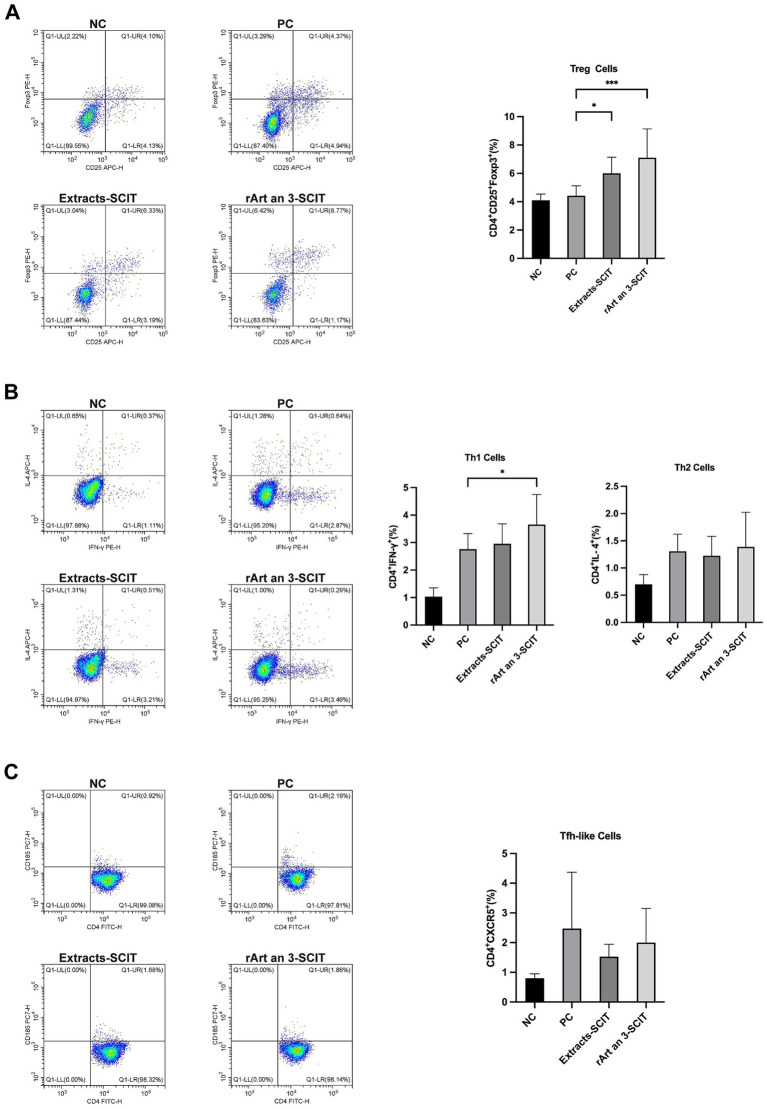
Representative flow-cytometry plots and pooled data are shown for splenic CD4 + T-cell subsets in each group (NC, PC, Extracts-SCIT, and rArt an 3-SCIT). **(A)** CD4^+^CD25^+^Foxp3^+^ regulatory T cells (Treg). **(B)** IFN-γ^+^ Th1 and IL-4^+^ Th2 cells within the CD3^+^CD4^+^ T-cell population. **(C)** CD4^+^CXCR5^+^ Tfh-like cells. Data are presented as mean ± SD (*n* = 10 per group). Statistical significance was determined by one-way ANOVA followed by Tukey’s multiple comparisons test. * *p* < 0.05, ** *p* < 0.01, *** *p* < 0.001 vs. PC group.

Flow cytometric analysis was performed to evaluate splenic CD4^+^ T cell subsets following SCIT (Figure 5). Both SCIT regimens significantly increased the proportion of Treg cells compared with PC mice (*p* < 0.05; Figure 5A), with numerically higher levels observed in the rArt an 3-SCIT group. Among effector CD4^+^ T cells, Th1 cells were elevated in both treatment groups, with a significant increase observed in the rArt an 3-SCIT group compared with PC mice (*p* < 0.05; Figure 5B). Th2 cell proportions did not differ significantly among the PC and SCIT-treated groups.

For Tfh-like cells (Figure 5C), PC mice exhibited higher frequencies than NC mice. Following SCIT, Tfh-like cell proportions were numerically reduced in both treatment groups; however, no statistically significant differences were detected among groups.

## Discussion

Most AIT research and clinical practice to date has focused on crude allergen extracts, whose complex composition and batch-to-batch variability constrain potency comparability and complicate mechanistic dissection (32, 33). Advances in molecular allergology now allow recombinant single allergens to be used in place of crude extracts for specific immunotherapy, providing more defined tools to probe key regulatory mechanisms (34). However, in Artemisia pollen–related asthma, the immunological properties of Art an 3 as a therapeutic antigen and its direct comparison with extract-based AIT have remained largely unexplored (35, 36). In this study, we systematically compared rArt an 3-SCIT with crude extract SCIT in the same *A. annua* pollen–induced asthma model. Overall, both regimens improved airway inflammation and AHR, while exhibiting different immunological tendencies related to inflammatory suppression and regulatory/Th1-associated immune changes. Crude extract more effectively suppressed IL-5/IL-17A and eosinophilic inflammation, whereas rArt an 3 was associated with relatively more evident IL-10, Th1, Treg and sIgG1 responses. These findings support Art an 3 as a molecularly defined recombinant antigen candidate with extract-comparable protective effects in this model, while also suggesting distinct immunological features between recombinant antigen-based and extract-based SCIT.

In interpreting these findings, the recombinant protein format should also be considered. Because Art an 3 is a small nsLTP of approximately 10 kDa, a SUMO fusion expression system was used to support recombinant protein production and purification. SUMO fusion is a commonly used recombinant protein production strategy that can improve expression yield, solubility, and purification efficiency, particularly for difficult-to-express proteins (31, 37). In the present study, IgE immunoblotting showed that the recombinant fusion protein retained IgE-binding reactivity, suggesting preservation of at least part of the IgE-recognized antigenic epitopes. Therefore, while the SUMO fusion format supported recombinant protein preparation for this study, the immunological findings should be interpreted cautiously in the context of this recombinant protein format.

Both SCIT regimens reduced peribronchial/perivascular inflammatory infiltrates and goblet-cell hyperplasia and lowered methacholine-induced Penh, indicating attenuation of airway inflammation and hyperresponsiveness in this model. Histological scoring suggested slightly greater improvement with Extracts-SCIT than with rArt an 3-SCIT, whereas AHR endpoints did not differ significantly between the two treatments, implying broadly comparable overall protective effects. BALF data further supported this interpretation: eosinophil counts were elevated in PC mice and were reduced by both SCIT regimens, with statistically significant suppression observed in the Extracts-SCIT group. Taken together, these observations indicate that both approaches attenuated airway inflammation, although eosinophil suppression was more evident with Extracts-SCIT. Nevertheless, both regimens achieved improvement in lung pathology and airway responsiveness, providing a basis for comparing their distinct immunological response patterns.

At the humoral level, both SCIT strategies reduced sIgE relative to PC, with a statistically significant decrease only in the Extracts-SCIT group, whereas rArt an 3-SCIT produced a non-significant downward trend and sIgE levels remained above those of NC. In contrast, allergen-specific IgG responses increased in both treatment groups, with a more evident increase in sIgG1 following rArt an 3-SCIT. Allergen-specific IgG responses have been implicated in AIT-associated immune protection, particularly through their potential to interfere with IgE–allergen interactions and FcεRI-dependent effector-cell activation (38, 39). In clinical AIT studies, increases in allergen-specific IgG, especially IgG4, and IgE-inhibitory activity have been associated with therapeutic benefit and sustained immune modulation (40–42). In the present study, the increased sIgG1 response after rArt an 3-SCIT is compatible with humoral immune modulation during AIT. However, because functional assays such as BAT, FAB, or IgE-inhibition assays were not performed, the actual IgE-blocking or effector-cell inhibitory capacity of sIgG1 was not directly demonstrated. Therefore, the increase in sIgG1 should be interpreted as a serological change associated with AIT-related immune modulation rather than direct evidence of functional blocking antibody activity. By contrast, the more pronounced sIgE decline observed with crude extract may relate to its higher antigenic complexity and broader epitope repertoire. Together, these findings suggest that Extracts-SCIT and rArt an 3-SCIT may engage distinct humoral response profiles, characterized, respectively, by stronger sIgE suppression and more evident sIgG1 induction. This divergence is not inconsistent with previous studies of allergoid or single-allergen AIT, in which comparable overall efficacy may be accompanied by different allergen-specific antibody response profiles (43–45). Nevertheless, the functional antibody activity associated with these serological changes requires further validation.

Cytokine profiles and BALF cell counts further highlight different immunological patterns between the two SCIT regimens. In PC mice, IL-5 and IL-17A were elevated, indicating a mixed Th2/Th17-associated inflammatory response. Both SCIT regimens reduced these cytokines, with more evident suppression observed in the Extracts-SCIT group. This was accompanied by a significant decrease in BALF eosinophils in the Extracts-SCIT group, whereas total leukocyte, neutrophil, and mononuclear cell counts showed only modest, non-significant declines. These findings suggest that crude extract more effectively attenuated IL-5/IL-17A-associated inflammatory responses in this model, which is consistent with previous reports showing that extract-based or multi-component allergen preparations can exert broader effects on inflammatory cytokine responses (25, 43). In contrast, rArt an 3-SCIT was associated with increased IL-10 and IFN-*γ*, suggesting a regulatory/Th1-associated immune pattern. Overall, Extracts-SCIT showed a more evident effect on suppressing inflammatory mediators and eosinophilic inflammation, whereas rArt an 3-SCIT was associated with regulatory/Th1-related immune changes; despite these different patterns, both regimens ultimately improved lung pathology and airway responsiveness.

At the T-cell subset level, both SCIT regimens were associated with changes in splenic CD4^+^ T-cell profiles. rArt an 3-SCIT showed significant increases in Th1 and Treg cells relative to PC, whereas Extracts-SCIT also increased Treg cells but showed a less evident effect on Th1 cells. In contrast, neither SCIT regimen produced a significant suppression of splenic Th2 cell frequencies at this time point. This pattern is consistent with the temporal dynamics of AIT, in which regulatory and Th1-associated changes may occur before stable downregulation of the Th2/IgE axis becomes evident (3, 46–49). Accordingly, the lack of a marked reduction in splenic Th2 cell frequencies at this time point is not unexpected.

In terms of differential effects, Extracts-SCIT showed stronger suppression of the Th2/IL-5/IL-17A-associated inflammatory axis, consistent with more evident attenuation of inflammatory drivers. In contrast, rArt an 3-SCIT was associated with relatively stronger Th1 and Treg responses, suggesting a distinct regulatory/Th1-associated immune pattern that may reflect the immunological features of a molecularly defined recombinant antigen. These differences may relate to the inherent properties of the antigens. Crude extracts contain a broad spectrum of T-cell epitopes and multiple accompanying non-allergenic components (50), which may contribute to broader and earlier modulation of inflammatory cytokine responses. In contrast, a single recombinant antigen has a more defined epitope composition and may provide more focused antigenic stimulation, such that downregulation of the Th2/IgE-associated inflammatory axis may be less pronounced during the current treatment window. This interpretation is consistent with the stepwise nature of AIT, in which regulatory and Th1-associated changes may occur before more stable suppression of Th2/IgE responses becomes evident (51, 52). Accordingly, the distinct immune response patterns observed here may reflect differences in antigenic complexity and immune activation context between crude extract-based and molecularly defined recombinant antigen-based SCIT.

Treg cells are central to AIT-associated peripheral immune tolerance. Through IL-10, TGF-β1 and CTLA-4, they can suppress effector T- and B-cell responses and contribute to immune regulation (46, 53). In our study, both SCIT regimens significantly increased Treg frequencies, with numerically higher levels observed in the rArt an 3-SCIT group. This was accompanied by increased IL-10, IFN-*γ* and sIgG1 in the rArt an 3-SCIT group. These observations suggest that rArt an 3-SCIT was associated with coordinated regulatory/Th1-related cellular changes and allergen-specific IgG responses. Previous studies have shown that IL-10-producing regulatory B cells may be associated with IgG4 responses and clinical benefit during AIT (54). However, Breg cells were not directly evaluated in the present study, and the functional inhibitory capacity of sIgG1 was not assessed. Therefore, the possible relationship among IL-10, regulatory cell responses and sIgG1 induction remains exploratory and should be further examined by B-cell phenotyping and functional antibody assays.

T follicular helper (Tfh) cells orchestrate germinal center (GC) responses, driving antibody affinity maturation and class switching, and the Tfh13 subset is critically involved in the generation of high-affinity IgE (55). In this study, we used splenic CD4^+^CXCR5^+^ cells as a Tfh-like population to reflect exploratory trends in peripheral humoral responses. However, this phenotype does not fully represent bona fide GC-Tfh cells, for which additional markers such as PD-1, Bcl6 and ICOS would provide more specific characterization. Tfh-like cells were numerically elevated in PC mice and numerically reduced after SCIT, particularly in the Extracts-SCIT group, although no statistically significant differences were detected among groups. In conjunction with the absence of further sIgE increases, the rise in sIgG1 and concomitant enhancement of Th1/Treg responses, these findings may reflect early changes in humoral immune regulation during the current treatment window. Given the role of GC-Tfh and related Tfh subsets in antibody affinity maturation and high-affinity IgE responses (56), more detailed characterization of GC-Tfh populations and longer treatment protocols may be required to determine whether this axis is further modulated by SCIT.

This study has several limitations. First, the increase in pollen-specific sIgG1 was assessed only at the serological level. We did not perform functional assays such as BAT, FAB, or IgE-inhibition assays; therefore, the actual IgE-blocking or effector-cell inhibitory capacity of sIgG1 was not directly demonstrated. Accordingly, any blocking-antibody-related interpretation in this study should be regarded as indirect and hypothesis-generating. Nevertheless, this serological change was observed in parallel with improvements in lung pathology and airway responsiveness, suggesting that it may be biologically relevant to the treatment-associated immune response, although its functional blocking activity remains to be demonstrated. Second, Tfh analysis relied on splenic CD4^+^CXCR5^+^ Tfh-like cells, which can capture exploratory trends in peripheral humoral responses but do not fully substitute for bona fide GC-Tfh cells. Additional markers such as PD-1, Bcl6, and ICOS would be required to more precisely characterize GC-Tfh responses; therefore, conclusions regarding GC activity and high-affinity IgE regulation remain indirect. Third, we did not further phenotype B-cell subsets such as Breg or GC-B cells, limiting mechanistic insight into the relationship among IL-10, regulatory cell responses, and allergen-specific IgG induction. Finally, this study did not include an alum-only control group; therefore, potential non-specific adjuvant-related effects cannot be fully excluded. Future studies incorporating functional antibody assays, more detailed GC-Tfh and B-cell phenotyping, alum-only controls, and extended immunotherapy protocols will be needed to further define the key checkpoints of Art an 3–mediated immune modulation.

## Conclusion

In this *A. annua* pollen–induced asthma model, rArt an 3-SCIT and Extracts-SCIT showed comparable protection against airway inflammation and AHR, while exhibiting distinct immunological response patterns. Extracts-SCIT more prominently attenuated eosinophilic inflammation and Th2/Th17-associated cytokine responses, whereas rArt an 3-SCIT was associated with more evident regulatory/Th1-related immune changes, including increased Treg/Th1 responses and *A. annua* pollen–specific sIgG1. These findings support rArt an 3 as a molecularly defined recombinant antigen candidate for Artemisia pollen AIT. Further studies incorporating functional antibody assays, detailed B-cell and GC-Tfh characterization, and extended treatment protocols are needed to validate the mechanisms underlying rArt an 3–based immunotherapy and to support its future translational development.

## Data Availability

The original contributions presented in the study are included in the article/Supplementary material, further inquiries can be directed to the corresponding author.
