# Supplementary material for: Art an 3 as a candidate recombinant antigen for Artemisia pollen immunotherapy: comparative efficacy and mechanistic insights
Source: Front Med (Lausanne). 2026 Jul 22;13:1864362. doi: 10.3389/fmed.2026.1864362 (PMC13438400; doi:10.3389/fmed.2026.1864362)
Supplement: Supplementary file 1 [file Data_sheet_1.pdf]

NOTE: Please save this file locally before filling in the table, DO NOT work on the file within your internet browser as changes will not be saved. Adobe Acrobat Reader (available free here) is recommended for completion.

# The ARRIVE guidelines 2.0: author checklist

## The ARRIVE Essential 10

These items are the basic minimum to include in a manuscript. Without this information, readers and reviewers cannot assess the reliability of the findings.

| Item | | Recommendation | Section/line number, or reason for not reporting |
|---|---|---|---|
| **Study design** | 1 | For each experiment, provide brief details of study design including:<br>a. The groups being compared, including control groups. If no control group has been used, the rationale should be stated.<br>b. The experimental unit (e.g. a single animal, litter, or cage of animals). | Reported in Methods — Sensitization, challenge and specific immunotherapy (SCIT) section, where four groups (NC, PC, Extracts-SCIT, rArt an 3-SCIT) are defined.<br>Reported in Methods — Sensitization, challenge and specific immunotherapy (SCIT) section; the experimental unit was a single mouse. |
| **Sample size** | 2 | a. Specify the exact number of experimental units allocated to each group, and the total number in each experiment. Also indicate the total number of animals used.<br>b. Explain how the sample size was decided. Provide details of any *a priori* sample size calculation, if done. | Reported in Methods — Sensitization, challenge and specific immunotherapy (SCIT) section (n=10 per group, 40 mice in total).<br>The sample size followed established murine allergy models where group sizes of ≈10 are considered sufficient to detect biologically meaningful effects while minimizing animal use |
| **Inclusion and exclusion criteria** | 3 | a. Describe any criteria used for including and excluding animals (or experimental units) during the experiment, and data points during the analysis. Specify if these criteria were established *a priori*. If no criteria were set, state this explicitly.<br>b. For each experimental group, report any animals, experimental units or data points not included in the analysis and explain why. If there were no exclusions, state so.<br>c. For each analysis, report the exact value of *n* in each experimental group. | Reported in Methods — Animals section (female BALB/c, 6–8 weeks, SPF-housed, healthy); no additional exclusion criteria were applied beyond the basic animal health and age requirements.<br>No animals or datapoints were excluded — all assigned mice completed assessment.<br>Exact n values are reported within figure legends and Results text. |
| **Randomisation** | 4 | a. State whether randomisation was used to allocate experimental units to control and treatment groups. If done, provide the method used to generate the randomisation sequence.<br>b. Describe the strategy used to minimise potential confounders such as the order of treatments and measurements, or animal/cage location. If confounders were not controlled, state this explicitly. | Reported in Methods — Sensitization, challenge and specific immunotherapy (SCIT) section (animals were randomly assigned to groups).<br>Procedural consistency was maintained (animals were handled in a constant cage order across sessions), but no formal confounder balancing strategy was implemented; this is acknowledged as a minor limitation, although cage order and |
| **Blinding** | 5 | Describe who was aware of the group allocation at the different stages of the experiment (during the allocation, the conduct of the experiment, the outcome assessment, and the data analysis). | Outcome assessment and data analysis were not blinded; no blinding was performed, which is acknowledged as a methodological limitation. |
| **Outcome measures** | 6 | a. Clearly define all outcome measures assessed (e.g. cell death, molecular markers, or behavioural changes).<br>b. For hypothesis-testing studies, specify the primary outcome measure, i.e. the outcome measure that was used to determine the sample size. | Reported in Methods — including AHR testing, histopathology scoring, BALF cell analysis, cytokines, serum antibodies, and T-cell subset profiling.<br>AHR and histopathology were treated as primary endpoints — implicit in Methods sections Assessment of airway hyperresponsiveness and Histopathology. |
| **Statistical methods** | 7 | a. Provide details of the statistical methods used for each analysis, including software used.<br>b. Describe any methods used to assess whether the data met the assumptions of the statistical approach, and what was done if the assumptions were not met. | Reported in Methods — Statistical analysis section (GraphPad Prism 10.0.2; ANOVA, Kruskal-Wallis, two-way ANOVA, normality and variance testing).<br>Reported in Statistical analysis section (Shapiro–Wilk and Levene tests applied; alternative nonparametric tests used if assumptions not met). |
| **Experimental animals** | 8 | a. Provide species-appropriate details of the animals used, including species, strain and substrain, sex, age or developmental stage, and, if relevant, weight.<br>b. Provide further relevant information on the provenance of animals, health/immune status, genetic modification status, genotype, and any previous procedures. | Reported in Methods — Animals section (female BALB/c, 6–8 weeks, 16–20 g).<br>Reported in Methods — Animals (SPF facility, purchased from Beijing SPF Biotechnology Co., Ltd.; ethical approval QDAE202309280001). |
| **Experimental procedures** | 9 | For each experimental group, including controls, describe the procedures in enough detail to allow others to replicate them, including:<br>a. What was done, how it was done and what was used.<br>b. When and how often.<br>c. Where (including detail of any acclimatisation periods).<br>d. Why (provide rationale for procedures). | All procedures fully detailed throughout Methods — including allergen sensitization, intranasal challenges, SCIT dosage, plethysmography, BALF processing,<br>SCIT dosing schedule and challenge timeline described in Methods — Sensitization, challenge and specific immunotherapy (SCIT) section.<br>Housing and acclimatization conditions reported in Methods — Animals section.<br>The rationale for SCIT comparison explained in Introduction, and procedural rationale implicit in Methods. |
| **Results** | 10 | For each experiment conducted, including independent replications, report:<br>a. Summary/descriptive statistics for each experimental group, with a measure of variability where applicable (e.g. mean and SD, or median and range).<br>b. If applicable, the effect size with a confidence interval. | Reported in Results — summary data provided in text and figures as mean ± SD.<br>Effect size and CIs were not reported, as formal interval estimation was not applicable to this dataset |
