# Supplementary material for: Art an 3 as a candidate recombinant antigen for Artemisia pollen immunotherapy: comparative efficacy and mechanistic insights
Source: Front Med (Lausanne). 2026 Jul 22;13:1864362. doi: 10.3389/fmed.2026.1864362 (PMC13438400; doi:10.3389/fmed.2026.1864362)
Supplement: Supplementary file 2 [file Data_sheet_2.pdf]

Endotoxin testing of purified recombinant Art an 3

Endotoxin contamination in the purified recombinant Art an 3 preparation was evaluated using a gel-clot Limulus amebocyte lysate (LAL) assay. Briefly, the LAL reagent was reconstituted with 100 μL bacterial endotoxin test water. Then, 100 μL of negative control solution, positive control solution, or recombinant protein sample was added to the corresponding LAL reaction tube. The recombinant protein sample was tested at a concentration of 1 mg/mL, and the positive control was prepared using a 2 EU/mL endotoxin standard solution.

After gentle mixing, the reaction tubes were incubated vertically at 37°C for 60 min. Gel clot formation was assessed by slowly inverting the tubes by 180°.

The negative control and recombinant protein sample remained liquid, whereas the positive control formed a stable gel clot. Based on the assay sensitivity of 0.25 EU/mL, the endotoxin level in the purified recombinant protein preparation was below the assay detection limit (<0.25 EU/mL), corresponding to <0.25 EU/mg protein.

Negative control	Recombinant protein sample<br>(1 mg/mL)	Positive control<br>(2 EU/mL)
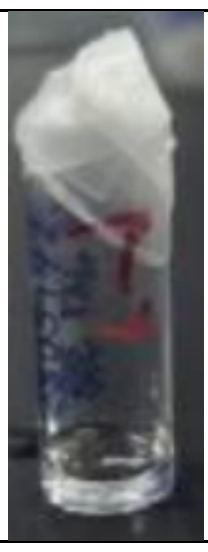	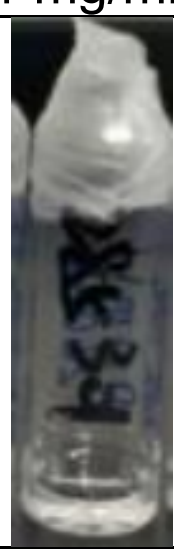	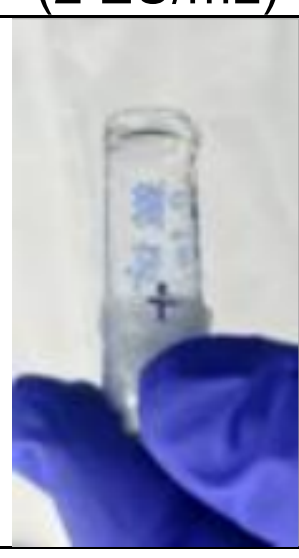
completely liquid	completely liquid	stable gel clot retained<br>after inversion
