# Supplementary material for: Art an 3 as a candidate recombinant antigen for Artemisia pollen immunotherapy: comparative efficacy and mechanistic insights
Source: Front Med (Lausanne). 2026 Jul 22;13:1864362. doi: 10.3389/fmed.2026.1864362 (PMC13438400; doi:10.3389/fmed.2026.1864362)
Supplement: Supplementary file 3 [file Data_sheet_3.pdf]

Flow cytometry gating strategy

Flow cytometry data were analyzed using FlowJo v10. Lymphocytes were first identified based on forward scatter and side scatter characteristics (FSC/SSC), followed by exclusion of cell aggregates using FSC-A/FSC-H gating. $CD4^+$ T cells were then selected as the parent population for downstream subset analysis.

For Treg analysis, Treg cells were defined as $CD25^+Foxp3^+$ cells within the $CD4^+$ T-cell population. For Tfh-like cell analysis, Tfh-like cells were defined as $CXCR5^+$ cells within the $CD4^+$ T-cell population. CXCR5 is also referred to as CD185 in the flow cytometry plots.

For intracellular cytokine analysis, $CD3^+CD4^+$ T cells were gated prior to assessment of IFN-γ and IL-4 expression. Th1 cells were defined as IFN-γ^+ cells, and Th2 cells were defined as IL-4^+ cells within the $CD3^+CD4^+$ T-cell population.

Representative gating plots are shown for each flow cytometry panel.

Treg

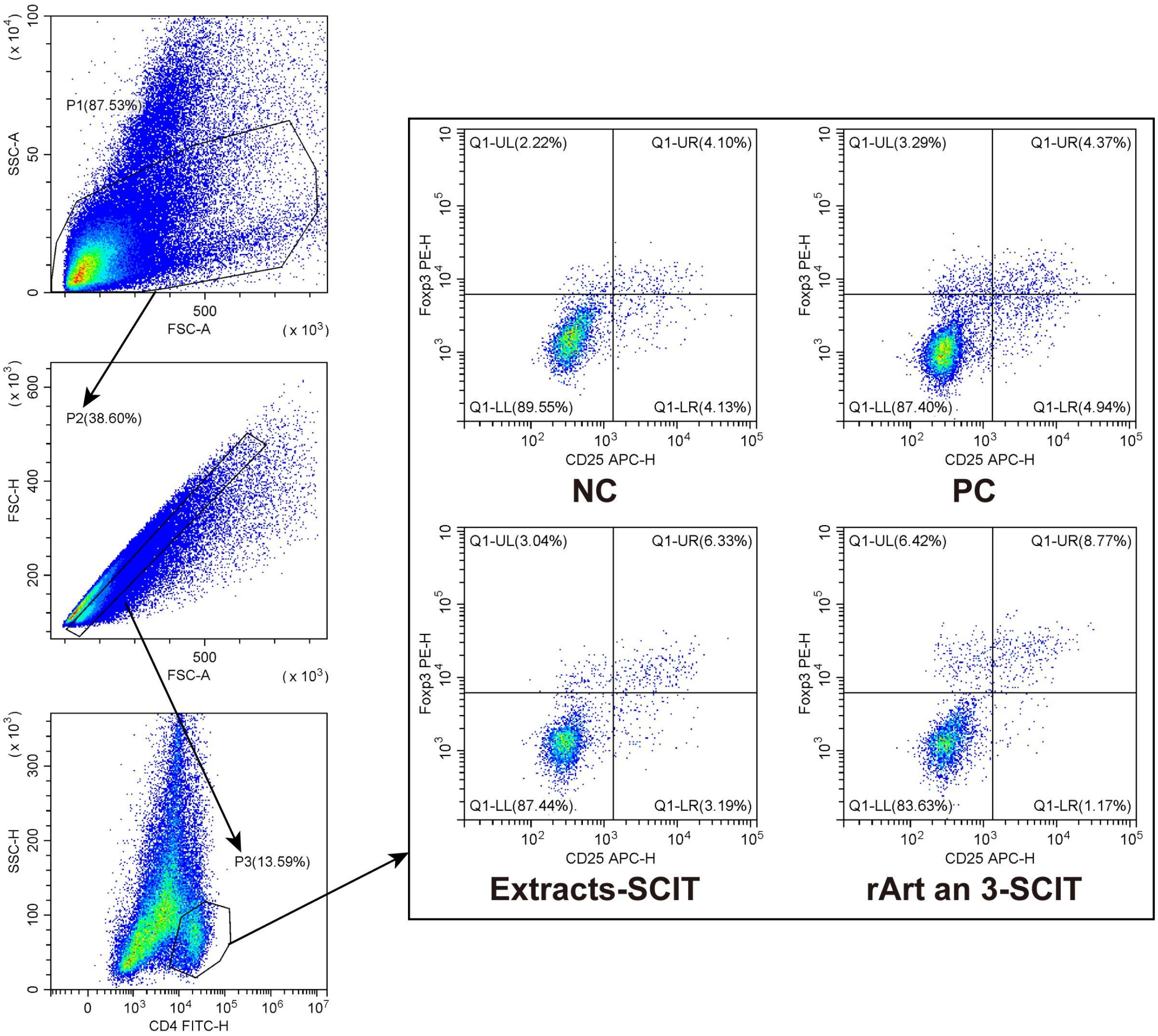

Th1 and Th2

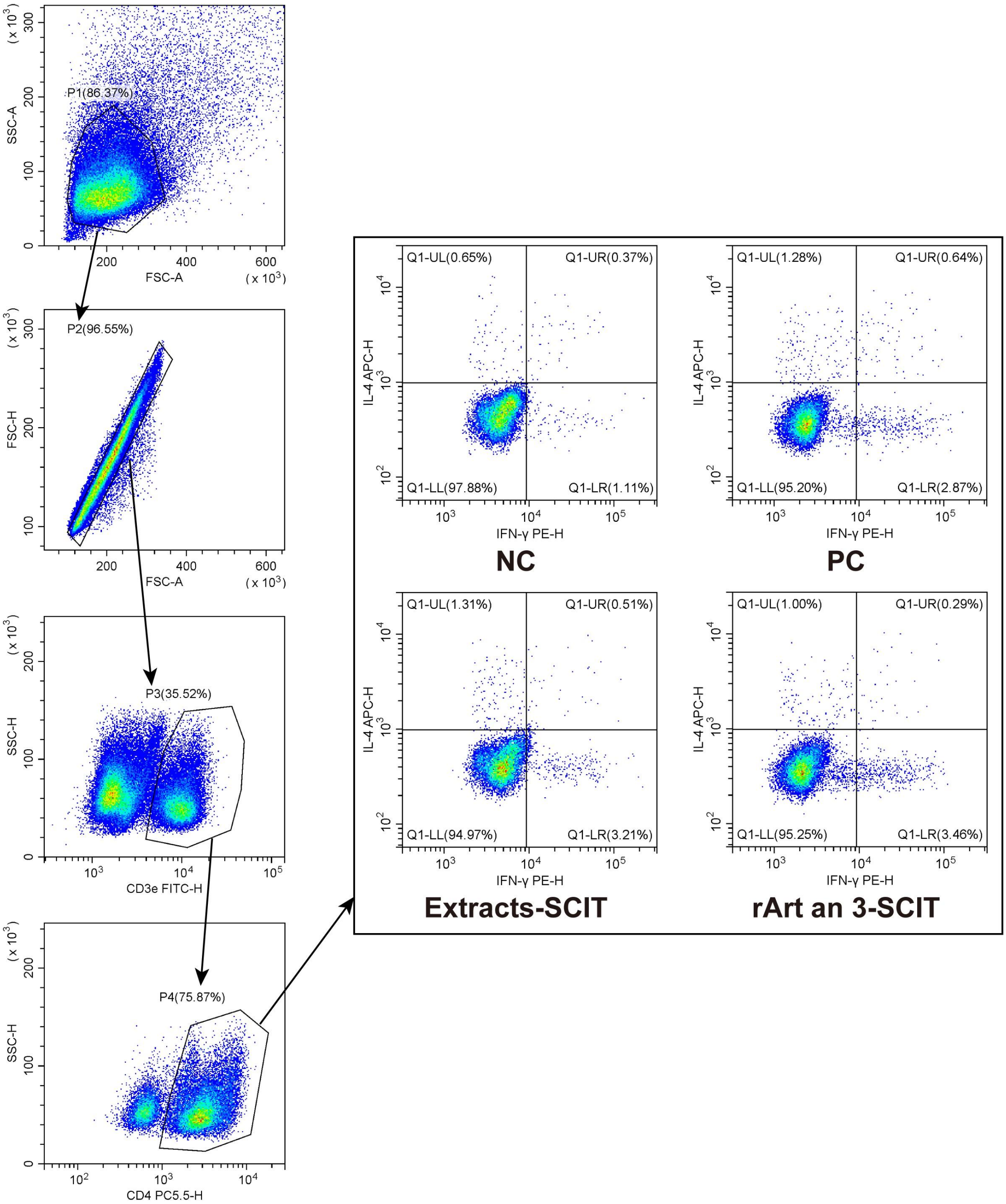

P1(86.37%)
SSC-A
FSC-A
P2(96.55%)
FSC-H
P3(35.52%)
SSC-H
CD3e FITC-H
P4(75.87%)
CD4 PC5.5-H
Q1-UL(0.65%)
Q1-UR(0.37%)
Q1-LL(97.88%)
Q1-LR(1.11%)
IL-4 APC-H
IFN-γ PE-H
NC
Q1-UL(1.28%)
Q1-UR(0.64%)
Q1-LL(95.20%)
Q1-LR(2.87%)
PC
Q1-UL(1.31%)
Q1-UR(0.51%)
Q1-LL(94.97%)
Q1-LR(3.21%)
Extracts-SCIT
Q1-UL(1.00%)
Q1-UR(0.29%)
Q1-LL(95.25%)
Q1-LR(3.46%)
rArt an 3-SCIT

Tfh-like

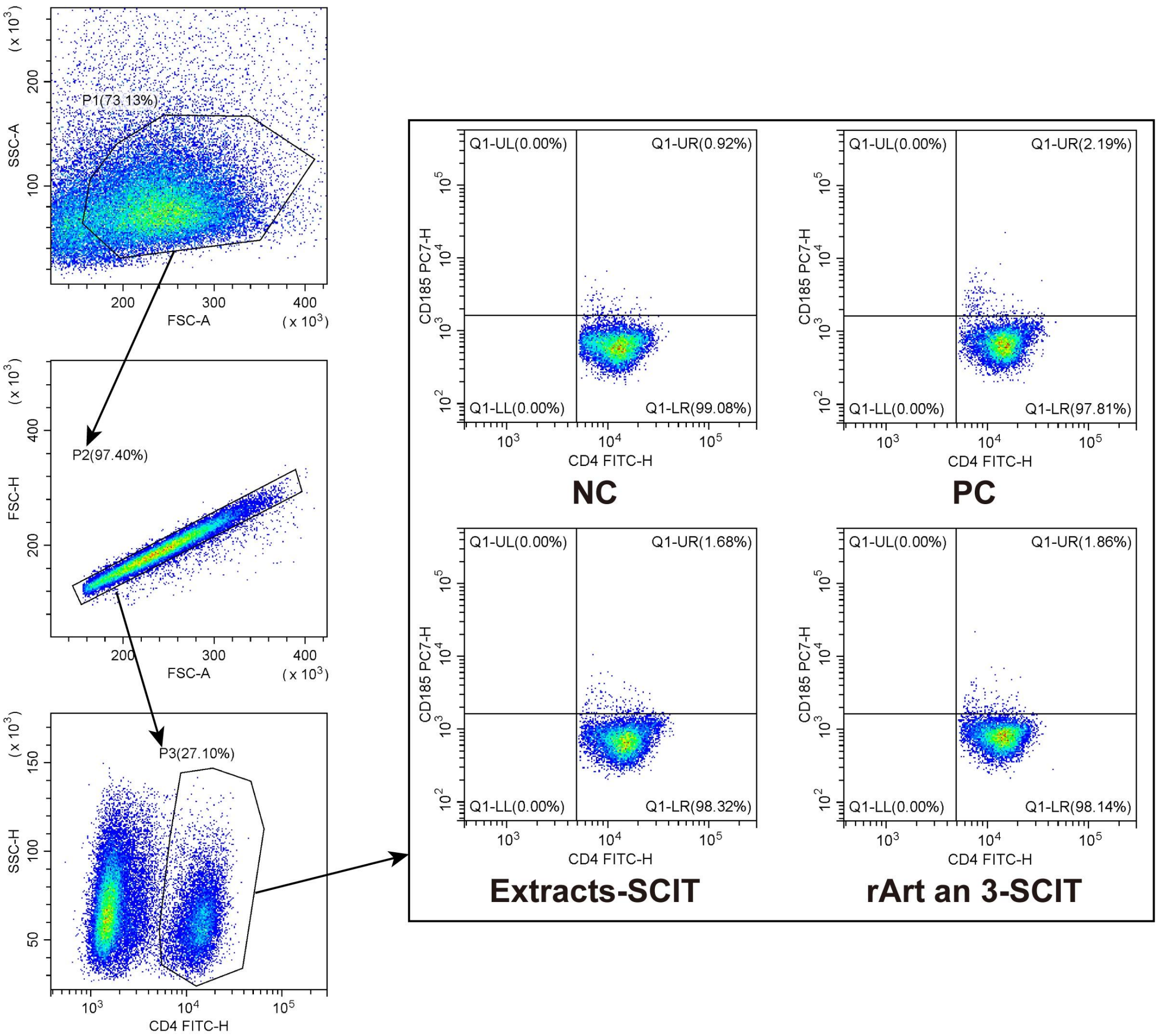

P1(73.13%)
SSC-A
FSC-A
P2(97.40%)
FSC-H
FSC-A
P3(27.10%)
SSC-H
CD4 FITC-H
Q1-UL(0.00%)
Q1-UR(0.92%)
Q1-LL(0.00%)
Q1-LR(99.08%)
CD185 PC7-H
CD4 FITC-H
NC
Q1-UL(0.00%)
Q1-UR(2.19%)
Q1-LL(0.00%)
Q1-LR(97.81%)
CD185 PC7-H
CD4 FITC-H
PC
Q1-UL(0.00%)
Q1-UR(1.68%)
Q1-LL(0.00%)
Q1-LR(98.32%)
CD185 PC7-H
CD4 FITC-H
Extracts-SCIT
Q1-UL(0.00%)
Q1-UR(1.86%)
Q1-LL(0.00%)
Q1-LR(98.14%)
CD185 PC7-H
CD4 FITC-H
rArt an 3-SCIT
